# Cancer chemopreventive potential of cooked glutinous purple rice on the early stages of hepatocarcinogenesis in rats

**DOI:** 10.3389/fnut.2022.1032771

**Published:** 2022-12-21

**Authors:** Huina Guo, Charatda Punvittayagul, Arpamas Vachiraarunwong, Warunyoo Phannasorn, Rawiwan Wongpoomchai

**Affiliations:** ^1^Department of Biochemistry, Faculty of Medicine, Chiang Mai University, Chiang Mai, Thailand; ^2^School of Basic Medical Sciences, Youjiang Medical University for Nationalities, Baise, China; ^3^Center of Veterinary Diagnosis and Technology Transfer, Faculty of Veterinary Medicine, Chiang Mai University, Chiang Mai, Thailand

**Keywords:** cancer chemoprevention, cooked purple rice, diethylnitrosamine, preneoplastic lesion, hepatocarcinogenesis

## Abstract

Cancer prevention using dietary phytochemicals holds great potential, particularly in the alternative treatment of liver cancer. Our previous study found that the methanol extract of cooked purple rice performed various biological functions including antioxidant, anti-inflammatory, and antimutagenic activities in *in vitro* assays. This study aimed to evaluate the chemopreventive effects of cooked glutinous purple rice extract (CRE) obtained from routine rice cooking method on diethylnitrosamine (DEN)-induced hepatic preneoplastic lesions in rats, along with its inhibitory mechanisms. CRE containing γ-oryzanols and high amounts of polyphenolic compounds, particularly cyanidin-3-glucoside, was fed to rats over a period 15 weeks. Additionally, injections of triple DEN at a concentration of 100 mg/kg BW were administered to rats once a week during the second, third, and fourth weeks of the experiment. The results revealed that CRE did not induce the formation of glutathione *S*-transferase placental form (GST-P) positive foci as a precancerous lesion during rat hepatocarcinogenesis, indicating non-carcinogenicity. Furthermore, CRE significantly reduced the number and size of GST-P positive foci in DEN-initiated rats. It also modulated microenvironment homeostasis by reducing the number of PCNA positive hepatocytes and by enhancing the number of apoptotic positive hepatocytes in the livers of DEN-initiated rats. Using RT-PCR analysis, CRE decreased the mRNA expression of some proinflammatory mediators, including interleukin-6, interleukin-1 beta, inducible nitric oxide synthase and cyclooxygenase 2, by attenuating the expression of cyclin E, the proliferation marker, while also inducing the expression of the apoptotic gene, Bcl2 associated X. The inhibitory mechanism at the early stages of hepatocarcinogenesis of CRE may be involved with the attenuation of cell proliferation, the enhancement of apoptosis, and the modulation of the proinflammatory system. Anthocyanins, flavonoids, and γ-oryzanol represent a group of promising chemopreventive agents in cooked glutinous purple rice extract. The outcomes of this study can provide an improved understanding of the potential role of the phytochemicals contained in cooked purple glutinous rice with regard to cancer alleviation.

## 1 Introduction

Liver cancer ranks sixth in terms of incidences of cancer worldwide and third in mortality rates, as has been estimated by Globocan 2020 (1). The risk factors of hepatocarcinogenesis are viral hepatitis, cirrhosis, alcohol consumption, metabolic syndrome, and aflatoxin B_1_ exposure (2). Even though liver resection, transplantation, and chemotherapeutic drugs are considered effective methods of treatment for liver cancer patients, prognosis of the disease is still poor (3). Importantly, the early screening methods for hepatocellular carcinoma (HCC), particularly those involving tumor markers, are not, in fact, precise (4, 5). Cancer chemoprevention has been reinforced as part of an effective strategy in the reduction of cancer incidences (6).

Nowadays, food intake is an important factor in cancer prevention, particularly in cancers that originate in the gastrointestinal biliary tract. Many investigations have reported that the regular intake of vegetables and fruits can be positively associated with the amelioration of the risks associated with developing liver cancer (7–9). Moreover, unsaturated fatty acids, vitamin E, vitamin B9, and β-carotene included in the human diet have been correlated with a reduction in HCC risk (8). Furthermore, many studies have shown that certain phytochemicals exhibited hepatoprotective effects and inhibited hepatocarcinogenesis through various mechanisms. Sasaki et al. proved that catechin-rich green tea extracts could protect mice against liver injuries with non-alcoholic steatohepatitis mediated by NFκB (10). Hesperidin obtained from *Citrus* species was found to prevent diethylnitrosamine (DEN)-induced hepatocarcinogenesis in rats by the upregulation of the Nrf2/ARE/HO-1 and PPARγ pathways and via a downregulation of the PI3K/Akt pathway (11, 12). Genistein present in soybeans suppressed carcinogenicity of DEN by inhibiting apoptosis and ameliorating inflammation via the AMPK pathway (13). Ginger extract and tomato extract have been reported for their ability to attenuate chemically-induced liver cancer development in rodents (14, 15). Furthermore, vanillic acid and protocatechuic acid effectively inhibited hepatic preneoplastic lesion formation in DEN and DMH induced rat hepatocarcinogenesis by utilizing detoxification system, while decreasing proliferation and enhancing apoptosis (16, 17).

Rice (*Oryza sativa* L.) has not only been acknowledged as a predominant source of energy for what is contained in its endosperm, but the bran and husk are also known to contain various bioactive compounds (18–20). Nowadays, colored rice has gained a significant amount of attention due to its high phytochemical contents in the form of anthocyanins, proanthocyanidins, phenolic acids, and flavonoids. This has resulted in the delivery of a wide range of beneficial activities that include antioxidant, anti-inflammatory, anti-diabetic, and anti-cancer activities (21). Cooking processes that employ heat can alter the relevant phytonutrient and phytochemical profiles of food. This is particularly true with regard to reductions in vitamin B complex and anthocyanins (22, 23). However, some compounds, such as vitamin E and gamma-oryzanols, can be released from the cellular matrix during heating (24). Our previous studies have reported that the methanol extract of cooked glutinous purple rice, containing both hydrophilic and low polar compounds, exhibited greater antioxidant, antimutagenic and anti-inflammatory activities using biochemical cell-based assays (25). To confirm the cancer chemopreventive activities of the hydrophilic components of cooked glutinous purple rice, this study aimed to evaluate the inhibitory mechanism of the methanol extract of cooked glutinous purple rice in the DEN-induced early stages of hepatocarcinogenesis in rats when compared with raw purple rice. Diethylnitrosamine (DEN) is a complete carcinogen that could induce cancer in various organs, particularly liver cancer. DEN-induced hepatocarcinogenesis in rats is one of the most well-known animal models which mimics human liver carcinogenesis at different stages of neoplastic transformation and progression. This model has been used to investigate the effects of chemopreventive or anticancer agents from natural products (26, 27).

## 2 Materials and methods

### 2.1 Reagents

Diethylnitrosamine (DEN) and 3,3′-diaminobenzidine tetrahydrochloride hydrate (DAB) were obtained from Sigma Aldrich (St. Louis, MO, USA). Rabbit polyclonal GST-placental form (GST-P) antibody was purchased from MBL (Nagoya, Japan). Mouse monoclonal proliferating cell nuclear antigen (PCNA) antibody was obtained from BioLegend (San Diego, CA, USA). Envision™ G/2 Doublestain System was purchased from Dako (Glostrup, Denmark). VECTASTAIN^®^ ABC Kit was obtained from Vector Laboratories (Burlingame, CA, USA). ApopTag peroxidase *In Situ* Apoptosis Detection Kit was acquired from Merck (Kenilworth, NJ, USA). All other chemicals and reagents used in the experiments were of analytical grade.

### 2.2 Sample preparation

*Oryza sativa* L., PIS 1 CMU, glutinous purple rice, was planted in Chiang Mai, Thailand, during August-November, 2020. Briefly, rice grains were soaked overnight in distilled water before being steamed in a rice cooker (1 L, MD, Thailand) for approximately 40 min. This procedure is a traditional method employed to obtain palatable cooked glutinous rice (25, 28). The lipophilic part of cooked rice was removed by soaking in dichloromethane over a period of 2 days. After separation, the remaining residue was macerated in methanol for 2 days in order to extract the hydrophilic components (29). The methanol filtrate was then evaporated with a rotary evaporator (Heidolph, MX07R-20-HD2E, Schwabach, Germany) under reduced pressure at 40°C and further freeze-dried with a bench top manifold freeze-dryer (Labconco FreeZone™, 2.5 L, Model 7740020, Kansas, MO, USA) at −50°C to obtain cooked glutinous purple rice extract (CRE). To compare its phytochemical profile and the chemopreventive activity with that of the uncooked rice, the raw glutinous purple rice extract (RRE) was prepared in a matched procedure. The glutinous purple rice extracts were kept at −20°C until being used.

### 2.3 Analysis of phytochemicals

The contents of the polyphenolic compounds and gamma-oryzanol derivatives of CRE and RRE were analyzed by high performance liquid chromatography (HPLC) (1260 Infinity series system, Agilent, Santa Clara, CA, USA). The procedures employed to measure some of the phenolic acids and anthocyanins were similar to those that have been described elsewhere (30). The anthocyanin standards were delphinidin-3-glucoside, cyanidin-3-glucoside, peonidin-3-glucoside, and malvidin-3-glucoside, while the phenolic acid standards were gallic acid, protocatechuic acid, 4-hydroxybenzoic acid, chlorogenic acid, syringic acid, *p*-coumaric acid, ferulic acid, ellagic acid, trans-cinnamic acid, and vanillic acid. Furthermore, the flavonoid and gamma-oryzanol contents were analyzed using the Zorbax C18 column sized 4.6 mm × 250 mm, 5 μm (Agilent Technologies, Santa Clara, CA, USA) as a stationary phase. Two systems of the mobile phase were used to detect the amounts of certain flavonoids. The first system involved a gradient elution of 1% acetic acid in water and 1% acetic acid in methanol as has been reported elsewhere (30) to detect the presence of catechins, epicatechins, rutin, isorhamnetin-3-glucoside, luteolin, and apigenin. In the second system, a gradient elution between 0.2% formic acid in water was used as solvent A and acetonitrile was used as solvent B in the mobile phase. While the percentage of solvent A declined from 85 to 44% within 23 min, 15% of solvent B increased from 15 to 56%. Quercetin-3-glucoside, quercetrin, myricetin, quercetin, tricin, and kaempferol were used as flavonoid standards. Additionally, the contents of gamma-oryzanol derivatives, including β-sitosteryl ferulate, campesteryl ferulate, cycloartenyl ferulate, and 24-methylene cycloartanyl ferulate, were measured by employing an isocratic elution system of 65% methanol and 35% acetonitrile for 45 min. The values of measured contents were expressed as mg per gram extract upon their amount.

### 2.4 Experimental animal models

Three-week-old male Wistar rats (90–100 g) were acquired from Nomura Siam International Co., Ltd., Bangkok, Thailand. All rats were granted free access to water and were fed a standard diet under a controlled temperature of 25 ± 1 °C with a 12-h light-dark cycle. After 1 week of acclimation, experiments were conducted according to the protocol approved by the Animal Ethics Committee, Faculty of Medicine, Chiang Mai University (53/2563).

**FIGURE 1:**
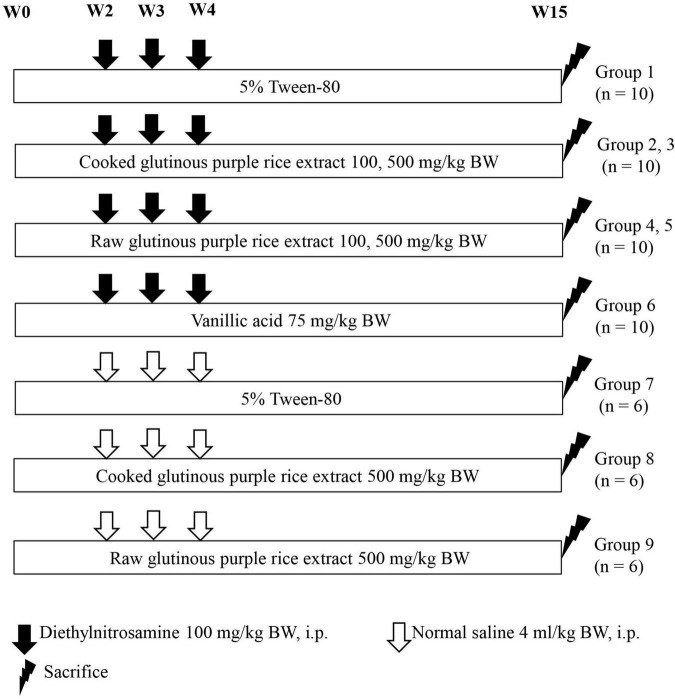
Experimental protocol employed to study the effects of cooked glutinous purple rice extracts on the early stages of rat hepatocarcinogenesis.

Fourth-week-old rats were randomly divided into nine groups, 10 rats per group for groups 1–6 and 6 rats per group for groups 7–9, as is shown in Figure 1. Groups 1–6 were intraperitoneally injected with 100 mg/kg body weight (BW) of DEN on weeks 2, 3, and 4 to induce hepatic preneoplastic lesion formation. The dose of DEN was used to initiate the early stage of rat hepatocarcinogenesis, according to our previous medium-term carcinogenicity studies (31, 32). Groups 7–9 were injected with a normal saline solution instead of DEN. Groups 1 and 7 were fed with 4 ml/kg BW of 5% Tween-80 as a positive and a negative control group, respectively. Groups 2 and 3 were fed 100 and 500 mg/kg BW of CRE, respectively. Groups 4 and 5 were administered with 100 and 500 mg/kg BW of RRE, respectively. The dosages of CRE and RRE was based on Dokkaew A et al. (32). Group 6 was orally administered with vanillic acid 75 mg/kg BW to represent a sample positive control group (16). Groups 8 and 9 were fed with a high dose of CRE and RRE, respectively, to determine their carcinogenicity. All rats were administered with the test compounds for 15 weeks. During the course of this study, food and water intake levels, as well as the body weights of the rats, were measured twice a week. All rats were euthanized by 4% isoflurane mixed with oxygen inhalation in a closed system for at least 5 min at the end of the experiment. Some vital organs including liver, spleen, and kidneys, were excised and weighed. Three pieces of liver tissues were fixed in 10% phosphate buffered formalin and embedded in paraffin wax. The 4 μm thick sections were used for immunohistochemistry study. The remaining liver portions were frozen in liquid nitrogen and then stored at −80°C for further analysis.

### 2.5 Immunohistochemistry

The detection of glutathione *S*-transferase placental form (GST-P) positive foci was performed by following the protocol described in our previous studies (31). Briefly, liver sections were deparaffinized in xylene and rehydrated in hydroalcoholic solvents. The endogenous peroxidase activity and non-specific binding protein were blocked by immerging the specimens in 3% H_2_O_2_ and 1% skim milk, respectively. The sections were subsequently incubated with polyclonal anti-rat GST-P antibody (1:1,000) for 2 h at room temperature. They were then incubated with goat anti-rabbit IgG secondary antibody conjugated with avidin-biotin peroxidase complex. A brown color appeared after specimens reacted with DAB. Finally, sections were counterstained with hematoxylin and the number and area of brown GST-P positive foci greater than 0.16 mm^2^ were measured under a light microscope using the LAS Interactive Measurement program version 4.10 (Leica Microsystems, Germany).

To determine the effect of CRE on cell proliferation, the immunohistochemical double staining of the proliferating cell nuclear antigen (PCNA) in GST-P positive foci and normal surrounding area was performed using an EnVision Doublestain system. The staining procedures were performed according to Khuanphram et al. (31). Liver sections were stained with anti-PCNA antibody (1:2,000 dilution) at 37°C for 1 h and anti-GST-P antibody (1:1,000 dilution) at 37°C for 1 h, following the manufacturer’s instruction. The numbers of PCNA positive hepatocytes in 2 mm^2^ areas of the GST-positive foci and surrounding areas were measured using a light microscope as has been described in previous studies (31).

### 2.6 TUNEL assay

Terminal deoxynucleotidyl transferase dUTP nick-end labeling (TUNEL) assay was employed to measure liver cell apoptosis. Liver slides were prepared and examined using an ApopTag^®^ Peroxidase *In Situ* Apoptosis Detection Kit according to the manufacturer’s instructions. Firstly, slides were deparaffinized, rehydrated, and pretreated with proteinase K and 3% H_2_O_2_. Then, the sections were incubated in equilibration buffer and terminal deoxynucleotidyl transferase (TdT), respectively. Afterward, they were treated with anti-digoxigenin antibody conjugated peroxidase and incubated with DAB to produce a desired color. The numbers of TUNEL-positive hepatocytes were counted under a light microscope and expressed as the number of apoptotic cells per area mm^2^.

### 2.7 RNA extraction and quantitative real-time PCR

Total RNA was extracted from rat livers using PureZol™ RNA isolation reagent (Bio-Rad, CA, USA). Accordingly, cDNA was synthesized from RNA using a high-capacity cDNA reverse transcription kit (Applied biosystems™, Foster City, CA, USA). The qPCR amplification was carried out using Sensi FAST™ SYBR Lo-ROX Kit (Bioline Reagent Ltd, London, UK) with specific primers (Table 1). The PCR conditions were set as follows; 95°C for 2 min, followed by 40 cycles at 95°C for 5 s, 56–60°C upon the specific primer for 10 s, and 72°C for 10 s (16). The related genes expression levels were normalized by β-actin and the expression fold change was calculated using the 2^–ΔΔct^ method.

**TABLE 1:** Primer lists for RT-PCR (32, 33).

Genes	5′-3′ primer sequence
*IL-1*β	Forward: 5′-CACCTCTCAAGCAGAGCACAG-3′ Reverse: 5′-GGGTTCCATGGTGAAGTCAAC-3′
*IL-6*	Forward: 5′-TCCTACCCCAACTTCCAATGC-3′ Reverse: 5′-TTGGATGGTCTTGGTCCTTAGCC-3′
*iNOS*	Forward: 5′-CAGGTGCTATTCCCAGCCCAACA-3′ Reverse: 5′-CATTCTGTGCAGTCCCAGTGAGGAA-3′
*COX2*	Forward: 5′-TGTATGCTACCATCTGGCTTCGG-3′ Reverse: 5′-GTTTGGAACAGTCGCTCGTCATC-3′
*Bax*	Forward: 5′-GTTGCCCTCTTCTACTTTGC-3′ Reverse: 5′-ATGGTCACTGTCTGCCATG-3′
*Cyclin E*	Forward: 5′-ATGTCCAAGTGGCCTACGTC-3′ Reverse: 5′-GGACGCACAGGTCTAAAAGC-3′
β*-actin*	Forward: 5′-ACAGGATGCAGAAGGAGATTAC-3′ Reverse: 5′-AGAGTGAGGCCAGGATAGA-3′

### 2.8 Statistical analysis

Results were expressed as mean ± SD values. A Student’s *t*-test was used to evaluate statistical differences between two groups of phytochemical content data. Multiple comparisons of animal experiment were analyzed by one-way analysis of variance (ANOVA) followed by the least significant difference (LSD) *post hoc* test. Differences were determined as significant at *p* < 0.05.

## 3 Results

### 3.1 Phytochemicals in glutinous purple rice

**FIGURE 2:**
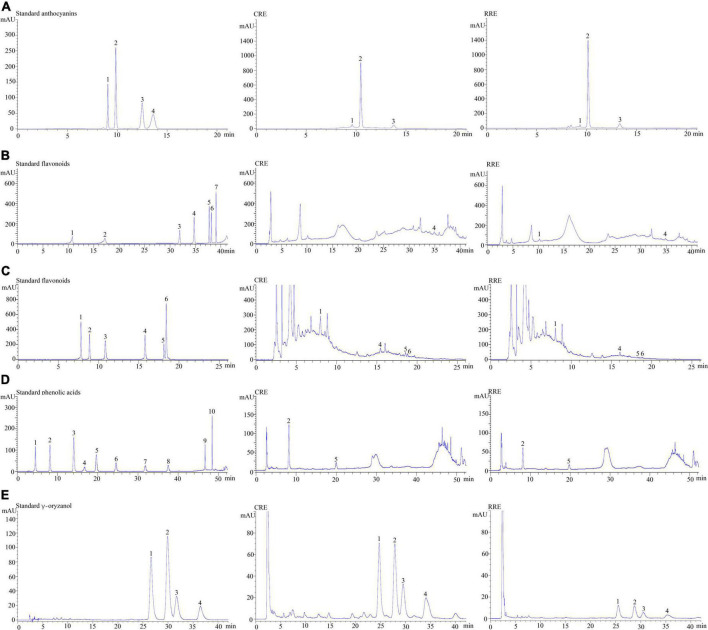
Chromatograms of some of the phytochemicals in glutinous purple rice. **(A)** Anthocyanins; peaks 1–4 represent delphinidin-3-glucoside, cyanidin-3-glucoside, peonidin-3-glucoside, and malvidin-3-glucoside, respectively. **(B)** Flavonoids; peaks 1–6 represent catechin, epicatechin, rutin, isorhamnetin-3-glucoside, luteolin, and apigenin, respectively. **(C)** Flavonoids; peaks 1–6 represent quercetin-3-glucoside, quercetrin, myricetin, quercetin, tricin, and kaempferol, respectively. **(D)** Phenolic acid; peaks 1–10 represent gallic acid, protocatechuic acid, 4-hydroxybenzoic acid, chlorogenic acid, syringic acid, p-coumaric acid, ferulic acid, ellagic acid, trans-cinnamic acid, and vanillic acid, respectively. **(E)** γ-Oryzanol; peaks 1–4 represent β-sitosteryl ferulate, campesteryl ferulate, cycloartenyl ferulate, and 24-methylene cycloartanyl ferulate, respectively. CRE, cooked glutinous purple rice extract; RRE, raw glutinous purple rice extract.

Table 2 illustrates the phytochemical contents of CRE when compared with raw glutinous purple rice extract (RRE). One hundred grams of dried uncooked and cooked glutinous purple rice yielded 0.80 ± 0.20 g of CRE and 1.88 ± 0.31 g of RRE, respectively. Anthocyanin compounds were found to be the chief hydrophilic constituents in glutinous purple rice variety Piesu 1 CMU. Cyanidin-3-glucoside was a major anthocyanin, while quercetin-3-glucoside and protocatechuic acid were observed to be the principle flavonoids and phenolic acids, respectively, in CRE. Furthermore, β-sitosteryl ferulate was a chief gamma-oryzanol derivative in CRE (Table 2). When compared with raw rice extract, there was a significant increase in most phytochemicals with the exception of cyanidin-3-glucoside, peodinin-3-glucoside, and catechins. The HPLC chromatograms for anthocyanins, flavonoids, phenolic acids, and gamma-oryzanol are illustrated in Figure 2.

**TABLE 2:** Phytochemical content in glutinous purple rice extracts analyzed by HPLC.

Compounds (per g extract)	Cooked glutinous purple rice extract	Raw glutinous purple rice extract
Cyanidin-3-glucoside (mg)	26.37 ± 0.10*	33.89 ± 0.07
Peonidin-3-glucoside (mg)	3.44 ± 0.09*	4.57 ± 0.07
Delphinidin-3-glucoside (mg)	1.00 ± 0.02*	0.46 ± 0.03
Quercetin-3-glucoside (mg)	1.47 ± 0.02*	0.74 ± 0.05
Quercetin (mg)	0.56 ± 0.02*	0.20 ± 0.00
Tricin (mg)	0.17 ± 0.00*	0.10 ± 0.00
Kaempferol (mg)	0.10 ± 0.00*	0.02 ± 0.00
Isorhamnetin-3-glucoside (mg)	0.06 ± 0.00	0.05 ± 0.00
Catechin (mg)	ND	0.28 ± 0.02
Protocatechuic acid (mg)	0.54 ± 0.00*	0.25 ± 0.00
Vanillic acid (mg)	0.10 ± 0.00*	0.07 ± 0.00
β-Sitosteryl ferulate (mg)	28.99 ± 1.05*	5.28 ± 0.02
Campesteryl ferulate (mg)	18.70 ± 0.82*	3.30 ± 0.04
Cycloartenyl ferulate (mg)	17.22 ± 1.05*	2.77 ± 0.11
24-methylene cycloartanyl ferulate (mg)	12.59 ± 0.38*	1.92 ± 0.04

Values are expressed as mean ± SD. ND, not detected.

*Significantly different from raw glutinous purple rice extract (*p* < 0.05).

### 3.2 Cooked glutinous purple rice inhibits hepatic preneoplastic lesion formation in rats

**FIGURE 3:**
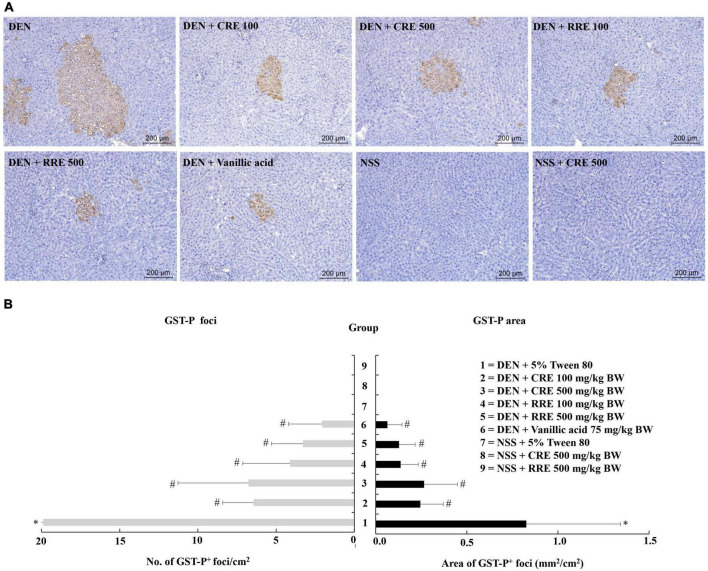
Effect of cooked glutinous purple rice extracts on hepatic GST-P positive foci formation in rats. **(A)** GST-P-positive foci in liver tissue, **(B)** number and area of GST-P-positive foci in rats. DEN, diethylnitrosamine; CRE, cooked glutinous purple rice extract; RRE, raw glutinous purple rice extract; BW, body weight. Values are expressed as mean ± SD. *Significantly different from the negative control group (group 7), *p* < 0.05. ^#^Significantly different from the positive control group (group 1), *p* < 0.05.

According to general observations, the administration of CRE or RRE did not affect the body weights, nor did it affect the amount of food and water intake of the rats in our experiments. However, injections of DEN repeated three times significantly reduced the body weights of the rats along with their food and water consumption indicating toxicity (Table 3). RRE was included in this study in order to compare the effects of cooking process on the cancer chemopreventive potential of glutinous purple rice. Furthermore, vanillic acid has been known to inhibit preneoplastic lesion formation in the livers of DEN-treated rats (16); therefore, it was employed as a positive anticarcinogen in our experiments. Glutathione *S*-transferase placental form (GST-P) positive foci is an established biomarker for the early stages of hepatocarcinogenesis in the livers of rats (34). However, GST-P positive foci was not detected in the livers of CRE or RRE treated rats indicating non-carcinogenicity (Figure 3). Furthermore, the formation of GST-P positive foci was found in all DEN-treated rats. Interestingly, the administration of 100 and 500 mg/kg BW of glutinous purple rice extracts prepared from cooked and uncooked rice significantly reduced the number and size of GST-P positive foci in the livers of DEN-initiated rats. This outcome was similar to the trend observed for the inhibitory effect of vanillic acid in this study. Notably, there were no significant differences for the preneoplastic attenuating formation that was observed in comparisons between cooked and raw purple rice. There is no dose-response effect in both CRE and RRE. It could be implied that the low dose of glutinous purple rice (Piesu 1 CMU variety) extracts was adequate to inhibit hepatic preneoplastic formation induced by DEN.

**TABLE 3:** General observations of medium-term carcinogenicity test on rats for 15 weeks.

Group	Chemical	Treatment	Body weight (g)		Consumption (/rat/day)	
			Initial	Final	Food (g)	Water (ml)
1	DEN	5% Tween 80	92 ± 6.3	401 ± 46.1*	17 ± 4.5*	24 ± 7.6*
2	DEN	CRE 100 mg/kg BW	92 ± 6.8	407 ± 39.4	19 ± 3.5	25 ± 11.3
3	DEN	CRE 500 mg/kg BW	93 ± 7.4	421 ± 28.6	20 ± 4.4	28 ± 9.6
4	DEN	RRE 100 mg/kg BW	92 ± 4.2	401 ± 23.4	18 ± 4.3	23 ± 8.6
5	DEN	RRE 500 mg/kg BW	92 ± 7.8	395 ± 28.0	20 ± 3.7	24 ± 6.8
6	DEN	VA 75 mg/kg BW	93 ± 8.7	441 ± 24.6	22 ± 4.9	28 ± 7.6
7	NSS	5% Tween 80	93 ± 13.3	467 ± 48.9	23 ± 6.0	31 ± 4.7
8	NSS	CRE 500 mg/kg BW	93 ± 4.2	457 ± 15.7	23 ± 4.8	30 ± 5.8
9	NSS	RRE 500 mg/kg BW	93 ± 4.1	453 ± 38.8	23 ± 5.3	29 ± 4.0

Values are expressed as mean ± SD.

*Significantly different from the negative control (group 7), *p* < 0.05.

CRE, cooked glutinous purple rice extract; RRE, raw glutinous purple rice extract; VA, vanillic acid; DEN, diethylnitrosamine; NSS, normal saline solution.

### 3.3 Cooked glutinous purple rice inhibits hepatocyte proliferation and promotes hepatocyte apoptosis in rats

**FIGURE 4:**
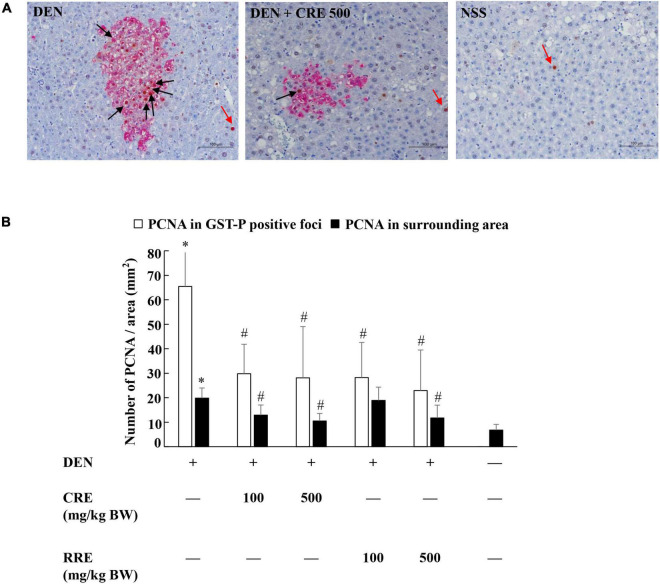
Effect of cooked glutinous purple rice extracts on cell proliferation in rat livers. **(A)** PCNA positive hepatocytes in liver tissue, **(B)** number of PCNA positive hepatocytes in GST-P positive foci and surrounding areas. Black arrow: PCNA-positive hepatocytes in GST-P positive foci and red arrow: PCNA-positive hepatocytes in the surrounding area. DEN, diethylnitrosamine; CRE, cooked glutinous purple rice extrac; RRE, raw glutinous purple rice extract. Values are expressed as mean ± SD. *Significantly different from the negative control group (*p* < 0.05). ^#^Significantly different from the positive control group (*p* < 0.05).

Proliferating cell nuclear antigen (PCNA) is a biomarker known to be able to indicate cellular proliferative potential. The number of PCNA-positive cells in the GST-P-positive foci was greater than in normal liver tissue (Figure 4A). The PCNA value was observed to significantly increase in the livers of DEN-treated rats. Furthermore, the oral administration of CRE at 100 and 500 mg/kg BW significantly reduced the number of PCNA labeling hepatocytes in both GST-P positive foci and in the surrounding areas of DEN-treated rats. The resulting outcomes were similar to those of the RRE treatment that was administered at high doses (Figure 4B). It was determined that CRE exhibited a stronger inhibitory effect on cell proliferation in carcinogen-treated rats than RRE.

**FIGURE 5:**
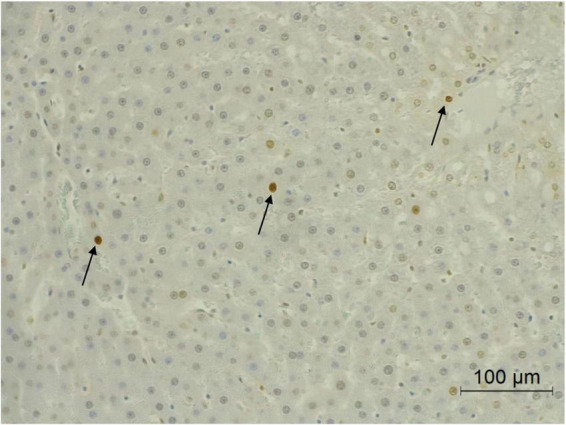
Apoptotic hepatocytes in liver tissue. Black arrow represents apoptotic hepatocytes.

Apoptosis in either normal or cancer cells play a crucial role on the regulation of cancer development. The apoptotic hepatocytes of this study were investigated by deoxynucleotidyl transferase dUTP nick end labeling (TUNEL) assay. Figure 5 illustrates the apoptotic hepatocyte in rats which was labeled as a brown hepatocyte. Under this animal condition, the apoptotic hepatocytes between DEN-treated rats and the vehicle control rats were not observed to be different. However, the administration of CRE and RRE in DEN-initiated rats markedly increased the number of apoptotic cells when compared with the group that had been given DEN alone (Table 4).

**TABLE 4:** Effect of cooked glutinous purple rice extracts on apoptotic hepatocytes in DEN-initiated rats.

Chemical	Treatment	TUNEL number/liver tissue (mm^2^)
DEN	5% Tween 80	1.74 ± 0.62
DEN	CRE 100 mg/kg BW	5.06 ± 0.81*
DEN	CRE 500 mg/kg BW	5.06 ± 0.58*
DEN	RRE 100 mg/kg BW	6.59 ± 1.54*
DEN	RRE 500 mg/kg BW	7.19 ± 2.56*
NSS	5% Tween 80	2.55 ± 0.67

Values are expressed as mean ± SD.

*Significantly different from group 1 (*p* < 0.05).

CRE, cooked glutinous purple rice extract; RRE, raw glutinous purple rice extract; DEN, diethylnitrosamine; NSS, normal saline.

### 3.4 Cooked glutinous purple rice down-regulates cell proliferation and inflammatory-involving genes but up-regulates apoptotic-involving genes in the livers of DEN induced-rats

**FIGURE 6:**
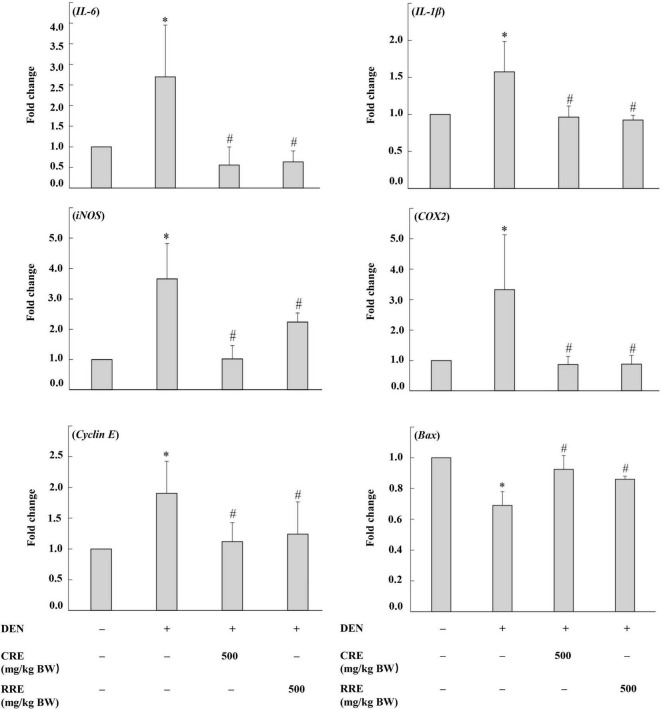
Effect of cooked glutinous purple rice extracts on some related gene expression levels of rats. DEN, diethylnitrosamine; CRE, cooked glutinous purple rice extract; RRE, raw glutinous purple rice extract. Values are expressed as mean ± SD. *Significantly different from the negative control group (*p* < 0.05). ^#^Significantly different from the positive control group (*p* < 0.05).

The imbalance of cell proliferation and apoptosis, as well as chronic inflammation, are known to be related to hepatocarcinogenesis. The evaluation of the expression of these apoptotic-involving genes was performed using real-time polymerase chain reaction (Figure 6). DEN promoted the expression levels of proinflammatory cytokines and enzymes, including interleukin 6 (*IL-6*), interleukin 1 beta (*IL-1*β), cyclooxygenase-2 (*COX2*), and inducible nitric oxide synthase (*iNOS*), in rat livers. The administration of CRE attenuated the expression levels of these proinflammatory markers in the livers of DEN-initiated rats. Moreover, *cyclin E*, which regulates cell cycles from the G1 to the S phase, was highly expressed in DEN-initiated rats. However, the treatment of CRE could reduce the levels of expression of *cyclin E* in rat livers. Meanwhile, the expression of the apoptotic *Bax* was observed to have been lowered in DEN treated rats, but this expression could have been modulated after CRE administration. Furthermore, the effects of the RRE treatment on the regulation of cell proliferation, as well as those of the apoptotic- and inflammatory-involving genes, were similar when compared to the effects caused by CRE.

## 4 Discussion

Fruits, vegetables, and whole grains are sources of micronutrients and phytochemicals that are known to be key in the relief of various chronic diseases. The consumption of rice has gradually increased among people due to elevated awareness of its nutritional benefits on the health and well-being of consumers. Purple rice is known to contain high amounts of vitamins and hydrophilic phytochemicals, particularly anthocyanins. Numerous studies have supported the contention that the bran and seeds, as well as the hull, exhibit cancer chemopreventive activities as has been determined by *in vitro* and *in vivo* models (29, 32, 35). Several studies have reported that the cooking process of rice could alter the phytochemical profile and biological activities of the finished product (24, 36, 37). Importantly, several studies have also reported that the household cooking process does alter the phytochemical contents in purple rice, while their biological activities, including their antioxidant, antimutagenic, and anti-inflammatory activities, may still persist (25, 38). Ti et al. found that the *in vitro* digestion process could improve the total phenolic content and antioxidant activities of cooked polished and brown rice, while some flavonoid content decreased significantly after the digestion process (39). This study primarily found that although the phytochemicals of glutinous purple rice were changed after the rice was cooked, it could lessen the preneoplastic formation in the livers of DEN-initiated rats when compared with uncooked rice.

DEN is a nitroso compound produced from the nitrosation of either the nitrates or nitrites found in certain foods, beverages, and tobacco smoke, with amino acids presented in the stomach. It has been widely used as a hepatocarcinogen in experimental rodents. DEN is mainly metabolized through CYP2E1 to ethyldiazonium ion, which then attacks DNA to form certain DNA adducts, such as O^6^-ethyl deoxyguanosine, and O^4^- and O^6^-ethyl deoxythymidine, resulting in carcinogenicity (40). Glutathione-*S*-transferase placental form is the one of glutathione-*S*-transferase isozymes which can be detected during embryo period and liver carcinogenesis (41). The administration of the cooked glutinous purple rice extract attenuated both number and size of GST-P positive foci in the liver of DEN-initiated rats. The lessening of this preneoplastic lesion in rats is associated with a reduction in the presence of the cellular proliferating marker, PCNA, particularly in the preneoplastic region, while increasing apoptotic hepatocytes. The possible inhibitory mechanism of cooked glutinous purple rice extract on the promotion stage of liver carcinogenesis may be involved with both the reduction of cell proliferation and the induction of apoptosis.

Cytokine release is a defense mechanism that restores cellular homeostasis during inflammation. The failure to regulate this process has been found to accelerate the transformation of precancerous cells to cancer cells (42). In this study, DEN promoted the release of some proinflammatory cytokines in rats, which was in line with previous reports (32). However, the inflammation is not the main hallmark of DEN-induced model in this study. Thus, the roles of the evaluated cytokines on preneoplastic lesion emergence should be further revealed. Furthermore, the activation of cell proliferation and the abatement of apoptosis by certain proinflammatory cytokines has resulted in the induction of hepatocarcinogenesis in rodents (43, 44). The administration of CRE attenuated the proinflammatory cytokine expression in the form of *IL-6, IL-1*β, *iNOS*, and *COX2*. IL-6 released from the liver inflammatory microenvironment plays a crucial role in promoting the proliferation and suppression of apoptosis by binding to its receptor. It has been reported that the activation of the STAT3 signal pathways mediated gene expression levels including those of certain proliferating genes, *Ras, Src*, and *cyclin D1*, as well as certain anti-apoptotic genes such as *Bcl-xL, Bcl-2*, and *P53* (45). Importantly, COX-2, as an inducible enzyme, catalyzed the conversion of arachidonic acid to prostaglandin E2 (PGE2), as has been demonstrated by the induction of apoptotic resistance in hepatocellular cells via the HIF-1α/PKM2 pathway (46). High levels of expression of *iNOS* produced greater amounts of NO by catalyzing the conversion of L-arginine to L-citrulline. Subsequently, NO reacted with ROS to form peroxynitrite causing various intracellular alterations such as oxidative stress, DNA damage, cytotoxic effects, and increased cell survival (47). Baker and colleagues have reported that IL-1β binding to the receptor IL-1RI may contribute to carcinogenesis through the activation of mitogen-activated protein kinase (MAPK) and nuclear factor-κB (NF-κB) pathways (48). We found that the amount of PCNA were markedly increased in preneoplastic lesion in the liver of DEN-initiated rats proving that excessive cell proliferation in this condition. Accordingly, our previous study found that DEN promoted the cell proliferation involved with the PI3K/Akt/mTOR pathway by proteomic analysis (49). After administration of CRE, the amount of PCNA was reduced concomitantly along with a reduction in the expression of cyclin E, a proliferating gene. Cyclin E binding to CDK 2 phosphorylated the retinoblastoma protein (Rb), thereby releasing E2F transcription factors to allow for E2F target gene expression while promoting the progression of G1 to S (50). Moreover, the cyclin E-CDK2 complex phosphorylated P21 and P27 allows cells to progress to the S phase. Thus, this would indicate that CRE could inhibit cell proliferation through reduction of the cell cycle from G1 to S by the attenuation of cyclin E expression.

The apoptotic pathway is capable of eliminating the damaged cells of an organism that could be modulated through proapoptotic and antiapoptotic proteins (51). Among these, Bcl-2-associated X protein (Bax) permeated the mitochondrial outer membrane facilitating cytochrome C release and activation of caspase, which could be a promising therapeutic target for the treatment of cancer (52). Accordingly, the administration of CRE reduced the number of apoptotic cells and enhanced the expression of the pro-apoptotic gene *Bax* in the livers of DEN-treated rats. This would imply that CRE could prevent hepatocarcinogenesis via the enhancement of the apoptotic pathway by increasing the expression of the Bax gene. Taken together, it can be suggested that CRE attenuates DEN-induced preneoplastic lesions in rats by inhibiting hepatocyte proliferation and promoting apoptosis through a reduction in the expression of proinflammatory cytokines mediators.

Many plant-based foods require heat transfer during the cooking process to lessen microbes or eliminate the presence of any anti-nutritional factors present in the raw form of the food substance such as trypsin inhibitors, amylase inhibitors, and goitrogens (53). However, it has been known that heating also destroys some of the nutritional value of food (54). Our previous studies have also found that the cooking process could alter and facilitate the release of some beneficial phytochemicals in glutinous anthocyanin-rich rice; whereas, its biological functions remain as has been determined by *in vitro* assays (25). Notably, the present study has confirmed the cancer chemopreventive activity of cooked glutinous purple rice during the early stages of hepatocarcinogenesis in rats. We found although some anthocyanins were reduced but their contents after cooking were still be the main phenolic compounds in glutinous purple rice. Cyanidin-3-glucoside has been reported to inhibit liver precancerous lesions in DEN and 2-AAF-induced rats via modulation of cell cycle progression (55). Furthermore, the amounts of flavonoids, phenolic acids, and gamma-oryzanol increased in cooked glutinous purple rice. It has been reported that these substances were involved in the transformation of certain parent phenolic compounds and the liberation of the phenolic compounds bound in the cellular matrix during the course of thermal induction (24, 56). Additionally, these compounds have been identified for their hepatoprotective effects in rodents. Gamma-oryzanol has been found to reduce DEN-induced hepatocellular carcinoma in Balb/C mice (57). Moreover, vanillic acid and protocatechuic acid contained in purple rice bran played a role in protecting rats from DEN-induced hepatocarcinogenesis (16, 17). Quercetin-3-glucoside acted as an antioxidant and anti-inflammatory agent by attenuating acetaminophen induced mice liver oxidative stress and inflammation via blockage of the NF-κB and MAPK pathways (58). Based on the above findings, it can be concluded that the main phenolic components of anthocyanins, γ-oryzanol, phenolic acids, and flavonoids were the potential cancer chemopreventive agents in CRE.

In this study, the compositions of the effective phytochemicals of cooked and raw glutinous purple rice were different, but the cancer chemopreventive activities were observed to be similar. However, high content of anthocyanins was observed in both cooked and raw glutinous purple rice extracts. It might be implied that the chemopreventive compounds in this purple rice was anthocyanin. Moreover, the effective doses of phytochemicals in glutinous purple rice of the Piesu 1 CMU variety which suggests as a high anthocyanin rice (59) is wide range that is responsible for its phytochemical content. The amount of CRE at 100 mg/BW/day in rats is equivalent to a human dose of 973 mg/day in a 60-kg person which can be daily obtained from domestic cooking of 128 g of a high anthocyanin rice. Thus, the regular consumption of anthocyanin-rich glutinous purple rice has therefore been recommended for its health benefits.

## 5 Conclusion

Phenolic compounds particularly cyanidin-3-glucoside and other phytochemicals including γ-oryzanol, have emerged as the potentially cancer chemopreventive constituents in cooked glutinous purple rice extracts. This anticarcinogenic action has been proven to impede the early stages of hepatocarcinogenesis in rats. Furthermore, the inhibitory mechanism might then be involved with the inhibition of cell proliferation and the enhancement of apoptosis, as well as the modulation of the inflammatory process. Accordingly, clinical studies on the cancer preventive potential of cooked glutinous purple rice should be conducted.

## Data availability statement

The original contributions presented in this study are included in the article/supplementary material, further inquiries can be directed to the corresponding author.

## Ethics statement

This animal study was reviewed and approved by Ethics Committee of the Faculty of Medicine, Chiang Mai University.

## Author contributions

RW: conceptualization. HG, AV, and WP: methodology. HG, CP, AV, and WP: formal analysis. HG and AV: investigation. HG, AV, and RW: data curation. HG: writing—original draft preparation. CP and RW: writing—review and editing. RW: supervision, project administration, and funding acquisition. All authors contributed to the article and approved the submitted version.
